# Relative Ratios Enhance the Diagnostic Power of Phospholipids in Distinguishing Benign and Cancerous Ovarian Masses

**DOI:** 10.3390/cancers12010072

**Published:** 2019-12-26

**Authors:** Tsukasa Yagi, Cyrus E. Kuschner, Muhammad Shoaib, Rishabh C. Choudhary, Lance B. Becker, Annette T. Lee, Junhwan Kim

**Affiliations:** 1 Feinstein Institutes for Medical Research, Northwell Health System, Manhasset, NY 11030, USA; tsuyagi-circ@umin.ac.jp (T.Y.); ckuschner1@pride.hofstra.edu (C.E.K.); mshoaib1@northwell.edu (M.S.); rchoudhary1@northwell.edu (R.C.C.); lance.becker@northwell.edu (L.B.B.); alee@northwell.edu (A.T.L.); 2 Donald and Barbara Zucker School of Medicine at Hofstra/Northwell, 500 Hofstra Blvd, Hempstead, NY 11549, USA

**Keywords:** lysophospholipids, LC-MS, diagnosis, lipidomics

## Abstract

Ovarian cancer remains a highly lethal disease due to its late clinical presentation and lack of reliable early biomarkers. Protein-based diagnostic markers have presented limitations in identifying ovarian cancer. We tested the potential of phospholipids as markers of ovarian cancer by utilizing inter-related regulation of phospholipids, a unique property that allows the use of ratios between phospholipid species for quantitation. High-performance liquid chromatography mass spectrometry was used to measure phospholipid, lysophospholipid, and sphingophospholipid content in plasma from patients with benign ovarian masses, patients with ovarian cancer, and controls. We applied both absolute and relative phospholipid ratios for quantitation. Receiver operating characteristic analysis was performed to test the sensitivity and specificity. We found that utilization of ratios between phospholipid species greatly outperformed absolute quantitation in the identification of ovarian cancer. Of the phospholipids analyzed, species in phosphatidylcholine (PC), lysophosphatidylcholine (LPC), and sphingomyelin (SM) were found to have great biomarker potential. LPC(20:4)/LPC(18:0) carried the greatest capacity to differentiate cancer from control, SM(d18:1/24:1)/SM(d18:1/22:0) to differentiate benign from cancer, and PC(18:0/20:4)/PC(18:0/18:1) to differentiate benign from control. These results demonstrate the potential of plasma phospholipids as a novel marker of ovarian cancer by utilizing the unique characteristics of phospholipids to further enhance the diagnostic power.

## 1. Introduction

Ovarian cancer has the highest mortality among gynecological cancers despite remarkable advances in the knowledge of molecular biology and treatment [1,2]. These poor outcomes are attributable to the lack of early presenting symptoms or reliable biomarkers, which limit physicians’ ability to establish an early diagnosis and initiate treatment. Currently, cancer antigen 125 (CA-125) represents the principle ovarian cancer biomarker; however, while it carries utility in assessing for cancer resurgence or tracking chemotherapy efficacy, it represents a poor screening tool, detecting approximately 50% of patients with stage I ovarian cancer. Furthermore, this marker alone is not recommended to distinguish between benign and malignant adnexal masses [3,4]. As such, pelvic examination and imaging tests are recommended for initial screening and the initial confirmation of an adnexal mass; however, these assessments carry similar inability to distinguish benign and malignant masses. Currently, the diagnosis of benign or malignant adnexal mass requires histopathologic examination of a surgically removed tumor [2].

An extensive effort has been made to advance physicians’ armamentarium for ovarian cancer detection, with the hope that finding a marker with improved sensitivity and specificity will facilitate earlier detection, treatment, and ultimately improve patient survival. Methods pursued thus far include proteomics, metabolomics, and lipidomics. However, the vast majority of recent research has investigated proteomics-based approaches [5]. While CA-125 and other protein-based biomarkers have demonstrated the most success detecting ovarian cancer thus far, recent studies have found that implementation of CA-125 in ovarian cancer screening protocols have not made an equivocal impact on patient survival. As such, no single marker has been implemented successfully to screen asymptomatic patients or assist in the distinction of benign and malignant ovarian pathology.

Metabolomics and lipidomics promise the potential to assist in ovarian cancer detection. This is achieved by characterizing the alterations to cellular metabolism associated with cellular transformation into cancerous processes [6]. Distinct metabolomic profiles have been extensively studied in animal and human models [7]. However, there has been less success translating these biomarkers towards consistent and clinically relevant tools. To this end, it is essential to explore the strengths that make phospholipid biomarkers promising for ovarian cancer surveillance and why recent literature has been unsuccessful in finding a consistent biomarker.

Phospholipidomics, including the study of lysophospholipids and sphingomyelin, has been relatively unrecognized as a potential approach towards identifying and characterizing ovarian pathology. Unlike other plasma lipid metabolites, most phospholipid species are stable with relatively lower within- and between-individual variations [8]. However, the use of phospholipids for ovarian cancer so far has not been satisfactory. While studies using global lipidomics approaches have reported different plasma phospholipids profiles in ovarian cancer patients, these findings were limited by unsatisfactory sensitivity and/or an absence of benign tumor [9,10,11,12]. Furthermore, the sole use of lysophosphatidic acid (LPA), the most intensively studied phospholipid, also has significant limitations due to the artificial increase during sample processing, storage, and analysis [13].

Phospholipid species are interrelated [14,15], as such, not only content changes in individual species but also their relative changes compared to other species are important for characterization of phospholipids [16,17]. This unique feature of phospholipids allows relative ratios between species within the same class as a quantitation, which provides a novel approach to identifying phospholipid biomarkers. The use of a second phospholipid species establishes an endogenous comparator, theoretically decreasing variability from factors such as sample quantity. Furthermore, more subtle differences in phospholipid profiles can be amplified by comparing phospholipids, which are elevated with those that are diminished. Finally, choosing to compare related phospholipid species allows the ratio to report on the status of a pathophysiologically relevant pathway. This represents an untouched area of exploration, which may supplement current proteomic and metabolomic biomarkers.

In the present work, we investigate the use of global lipidomics to identify phospholipid biomarkers with the potential to distinguish control, benign, and cancerous ovarian conditions. Using an established LC-MS method, we compared phospholipid levels in plasma samples obtained from patients with ovarian cancer, patients with benign ovarian tumors, and non-cancer controls. Phospholipids contents were analyzed and compared based on both absolute and relative phospholipid content. We found significant differences between the phospholipid profile in control, benign, and cancer groups. These differences were used to develop several phospholipid ratios capable of distinguishing normal, benign, and cancer groups. In this way, we demonstrate a novel approach to global lipidomics, validating the use of relative phospholipid content for biomarker analysis. Furthermore, we identify several promising phospholipid ratios which, with further validation, could improve ovarian cancer detection and differentiation of benign and cancer.

## 2. Results

### 2.1. LC-MS Analysis of Lysophospholipids

A representative total ion chromatogram and the mass spectra of phospholipids and lysophospholipids in control samples is shown in Figure S1. Their retention time and MS and MS/MS data compared to standard phospholipids identified the peaks [18]. The total ion chromatogram shows major phospholipids present in human plasma, phosphatidylethanolamine (PE), phosphatidylinositol (PI), phosphatidylcholine (PC), and sphingomyelin (SM). Abundant lysophospholipids, LPC and LPE, are also visible in the total ion chromatogram. PE contains a significant amount of plasmalogens, which contain an ether linkage at the sn-1 position (PEP). However, PC plasmalogens (PCP) species account for only a minor portion of PC.

A previous study showed PEP constituting up to half of the total PE in human plasma [19], which is consistent with our observations. The MS spectra shows that the PE, PC, and PI species contain common fatty acids, such as palmitic acid (16:0), stearic acid (18:0), oleic acid (18:1), linoleic acid (18:2), arachidonic acid (20:4), and docosahexaenoic acid (22:6). LPE and LPC also are constituted with these major fatty acids. SM contains myristic acid (14:0), arachidic acid (20:0), behenic acid (22:0), and nervonic acid (24:1). These are fatty acids commonly found in mammalian species, including humans. We also found unusual SM species such as SM(d18:2/18:0) and SM(d18:2/22:0) with a high abundance. We provide the MS/MS/MS spectrum of SM(d18:2/18:0) as an example to prove the structure of the species (Figure S2). The MS/MS/MS spectrum of SM(d18:1/16:0) is also included as a comparison Our analysis is focused on these major phospholipid species. Our analysis is focused on major phospholipid species containing these common fatty acids. We have also found less abundant phospholipids such as phosphatidylglycerol (PG) and phosphatidylserine (PS), but did not include them in this study due to their insufficient peak intensities, which may be inconsistently detected when interfered by more abundant ions. We also detected LPA and LPI, which we found to be unstable, resulting in exclusion from our study [13].

### 2.2. Phospholipids Content

We first compared the total content of individual classes of phospholipids and lysophospholipids by normalizing the peak areas to the areas of internal standards. We used PME as an internal standard for phospholipid species. The ratio can be directly converted to concentration using standard curve as previously shown [18]. LPE and LPC were normalized using LPE (17:1) and LPC (17:1), respectively.

**Figure 1:**
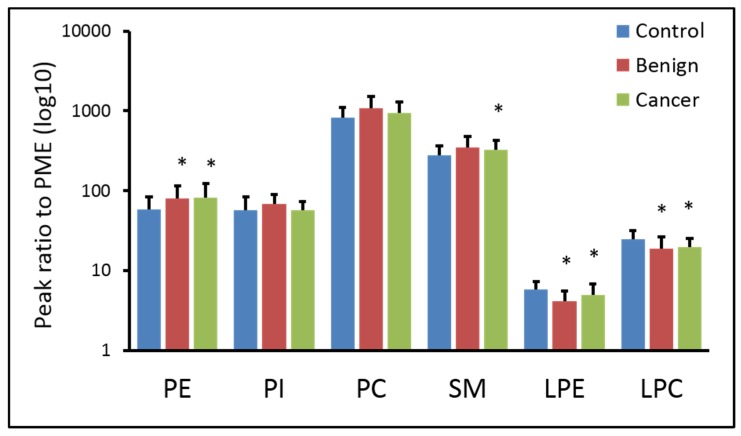
Content of individual classes of phospholipids and lysophospholipids. There is no major difference in the contents of plasma phospholipids (PME, internal standard; PE, phosphatidylethanolamine; PI, phosphatidylinositol; PC, phosphatidylcholine; SM, sphingomyelin; LPE, lysophosphatidylethanolamine; LPC, lysophosphatidylcholine; *, *p* < 0.05 against control).

Figure 1 shows the changes in the normalized total content of each class of phospholipids and lysophospholipids. The quantitation of PE and PC contains plasmalogens. We found that the total PE content is ~40% higher in benign and cancer compared to control. SM was higher by ~20% in benign and cancer. LPE is 30% lower in benign and 15% lower in cancer compared to baseline. LPC is ~20% lower in benign and cancer than control. Overall, phospholipids contents are generally higher in benign and cancer than control, whereas lysophospholipids contents are lower in benign and cancer. However, there is no significant difference in the content of phospholipids or lysophospholipids between benign and cancer.

**Figure 2:**
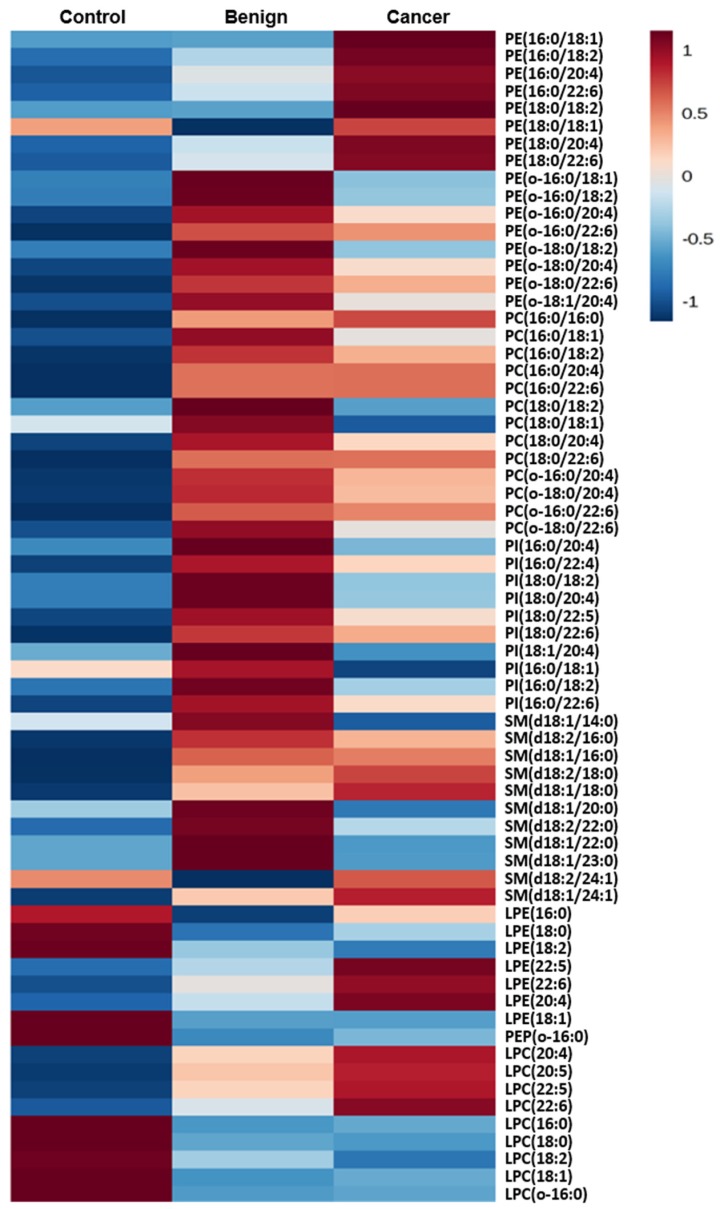
A heat map provides intuitive visualization of a difference in the phospholipid profile between the control, benign tumor, and ovarian cancer patients.

We then analyzed the changes in the content of individual phospholipids and lysophospholipids species. A heat map representation of phospholipid content clearly shows a difference in the content of individual phospholipids between the three groups, control, benign, and cancer (Figure 2).

PE species are generally higher in cancer plasma than control and benign, whereas PEP are higher in benign than in control or cancer. This result indicates that the increased total PE shown in Figure 1 may be due to increased diacyl PE in cancer, while it is mainly due to increased PEP in benign. Consistent with the trend found in Figure 1, all major PC and PI species are higher in benign than the other two groups (Figure 2). In SM, species are higher in benign than in control. Compared to cancer, SM species in benign are also higher except for SM (d18:2/18:0) and SM (d18:1/18:0). It is also noted that SM (d18:1/14:0) is dramatically lower. LPC and LPE species containing saturated fatty acids are higher in the control, whereas species containing polyunsaturated fatty acids (PUFAs) are higher in cancer. Overall, the heat map shows that phospholipid profiles are clearly different between patients in the control, benign, and cancer groups. However, this difference is not consistent by statistical analysis (Table 1). In particular, between benign and cancer, only PI (18:1/20:4), PE (16:0/18:1), PI (18:0/20:4), and PI (16:0/18:1) are found to be different with statistical significance.

As an alternative approach, we normalized the peak areas of individual phospholipids to the total content of phospholipids, where the total content of phospholipid serves as an endogenous internal standard. This approach is to identify individual species with the most significant change by reducing sample amount variations that exogenous internal standards cannot correct [20]. This approach is possible because changes in the total content of each class of phospholipid and lysophospholipid are mild to moderate (Figure 2). As shown in Table 2, we found more metabolites with a statistically significant difference. Various species in PC, PI, and PE are found to be different between control vs. benign and control vs. cancer, showing that ovarian cancer significantly alters phospholipid profiles in plasma. We also found difference between benign vs. cancer, particularly in SM and PE.

Between control and cancer, LPE and LPC—including LPEP and LPCP species—show the most significant differences. PC and SM were also found to be useful to differentiate cancer from control. In general, species containing long chain fatty acids (14 to 18 carbons) are lower in cancer; unlike species containing very long change fatty acids (>18 carbons). Between benign and cancer, PE and SM species show the most difference; PEP are lower in cancer, whereas PE are lower in benign. Our results show significant differences in the content of metabolites, substantiating the notion that phospholipid species are interrelated and there is a degree of regulation that exists in the tissues, which gets dysregulated during tumorigenesis. Although variations were significantly reduced by normalization to the total content of each class of phospholipids and some species displayed excellent sensitivity and specificity to distinguish between control and ovarian cancer, none of the species were able to be used to distinctly distinguish between benign, and benign and cancer.

### 2.3. Normalization to Other Species Within a Class

**Figure 3:**
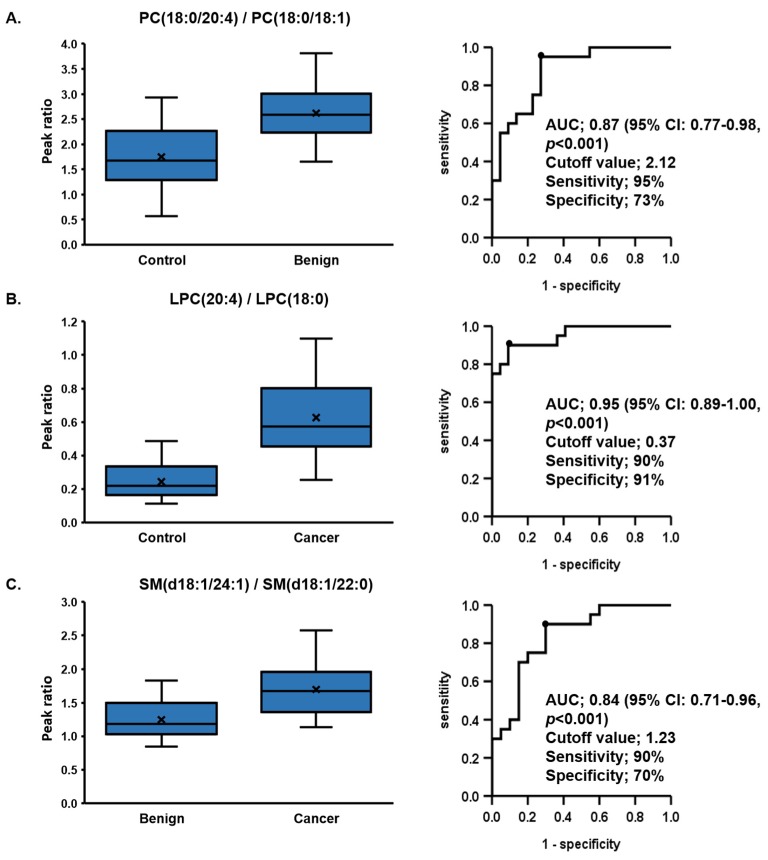
Receiver operating characteristic analysis of phospholipids ratios. (**a**) The area under the curve (AUC) of the capacity of the ratios of PC (18:0/20:4)/PC (18:0/18:1) to differentiate benign from control was 0.87, (**b**) the AUC of the capacity of the ratios of LPC (20:4)/LPC (18:0) to differentiate cancer from control was 0.95, and (**c**) the AUC of the capacity of the ratios of SM (d18:1/24:1)/SM (d18:1/22:0) to differentiate benign from control was 0.84. The intergroup difference was compared using the Mann–Whitney U Test.

We normalized a content of species to another species within the same class. In order to accomplish this, we chose species whose content was either increased in one group and decreased in another and determined the ratio between these two species. Using this approach, we identified multiple combinations that showed excellent separation between the 3 groups. For example, the ratio of SM (d18:1/24:1) was lower in cancer than in benign, but SM (d18:1/14:0) was higher in cancer. Therefore, the ratio of SM (d18:1/24:1)/SM (d18:1/22:0) was significantly high in cancer. In this way, we identified numerous phospholipid pairs, whose ratios are significantly different between the three groups. These pair markers were further analyzed by receiver operating characteristic (ROC) curve, the top five markers with best performance are shown in Table 3 and bar graphs and ROC curves of the best markers are shown in Figure 3 as an example.

Figure 3a shows the best area under the curve (AUC) of the ratios of PC(18:0/20:4)/PC(18:0/18:1) with the capacity to differentiate benign from control at 0.87 (95% CI: 0.77–0.98, *p* < 0.001) with a cutoff value of 2.12, a sensitivity of 95%, and a specificity of 73%. Figure 3b shows the best AUC of the ratios of LPC (20:4)/LPC (18:0) with the capacity to differentiate cancer from control at 0.95 (95% CI: 0.89–1.00, *p* < 0.001) with a cutoff value of 0.37, a sensitivity of 90%, and a specificity of 91%. Figure 3c shows the best AUC of the ratios of SM (d18:1/24:1)/SM (d18:1/22:0) with the capacity to differentiate benign from control at 0.84 (95% CI: 0.71–0.96, *p* < 0.001) with a cutoff value of 1.23, a sensitivity of 90%, and a specificity of 70%.

### 2.4. No Changes in the Ratio Occur When the Sample Amount is Altered

To demonstrate the utility of implementing relative phospholipid ratios, we examined the impact that variation in the quantity of sample collection carries on quantitation using a relative ratio. We show that when implementing a relative ratio, the species in each class of phospholipids and lysophospholipids have essentially the same response despite significant sample quantity variations (25 µL, 50 µL, or 100 µL) (Figure S2). LPC (22:6)/LPE (o-16:0) and SM (d18:1/16:0)/SM (d18:1)/22:0 shows moderate change with the rate of 0.2% /µL and 0.4% /µL, respectively. Even with 10% of pipet errors, the variation caused by sample amount variation will be less than 3%, which is insignificant. This establishes an essential component of clinical reliability in the described technique; that species ratio is unaffected by variance in sample collection.

## 3. Discussion

Our study provides support for the use of relative phospholipid quantitation in the quantitation of ovarian cancer biomarkers. Furthermore, we identified multiple phospholipid markers which, in our patient population, distinguished control vs. benign, control vs. cancer, and benign vs. cancer, with excellent sensitivity and/or specificity. Among those, LPE (22:6)/LPE (o-16:0) has the best sensitivity in distinguishing between control and benign, SM (d18:1/24:1)/SM (d18:1/22:0) has the best sensitivity between control and cancer, and PE (16:0/18:1)/PE (o-18:0/18:2) has the best specificity in distinguishing between benign and cancer. The existence of multiple markers, which can be quantified through the same LCMS protocol, facilitates the option to tailor biomarker selection based on the purpose of diagnosis, e.g., control vs. cancer or benign vs. cancer, without requiring additional testing. Moreover, multiple markers may provide a unique algorithm to differentiate potential ovarian masses. Future development of an algorithm that combines these ratios with higher accuracy may improve upon the predictive power of the previously described phospholipid ratios. Overall, our results demonstrate that phospholipids have a great potential to serve as novel diagnostic markers for ovarian cancer.

Despite substantial advances in understanding cancer pathology, the survival of ovarian cancer patients has not been significantly improved in the last 20 years. Time until disease discovery appears to play an essential role. While early discovery of local ovarian cancer carries a 90% survival, the majority of ovarian cancers are diagnosed at a late stage, which carries a 5-year survival less than 30%. Since the successful screening study shown by Petricoinet et al. [21], a wide variety of protein markers have been identified [22,23]. However, alongside an inability to demonstrate improved detection compared to CA-125, proteomics-based biomarkers require significant sample preconditioning to reduce interference from more abundant proteins [23,24]. In a recent Prospective Phase III trial utilizing the EPIC cohort, Terry et al. [25], analyzed the diagnostic value of four known or promising protein biomarkers, CA-125, HE4, CA-72.4, and CA-15.3, in 810 invasive ovarian cancer cases. Their findings further confirm that CA-125 remains the single best biomarker for early detection of ovarian cancer, with minimal improvement when combined with other protein biomarkers. These findings emphasize the need for a variety of ovarian cancer biomarkers, to avoid solely focusing on biomarkers which rely on CA-125, while also supplementing the diagnostic value of CA-125 in related expressive ovarian cancers. In this setting, our data clearly show the potential benefit in investigating alternative biomarkers to supplement CA-125.

Metabolomics provides a rich source of potential supplemental biomarkers. However, this approach carries two potential weaknesses. Metabolomics and proteomics provide a rich source of potential supplemental biomarkers, yet each carry inherited weaknesses. Metabolomics and proteomics are susceptible to variability based on confounding comorbidities and biomarker stability. Factors such as differences in age, gender, comorbid metabolic diseases, diet, and fasting status can significantly impact serum levels of metabolites and proteins [26,27]. Furthermore, metabolomics is particularly susceptible to degradation during sample processing [5]. However, previous research has demonstrated that phospholipids are less impacted by variability in diet and, with the exception of LPA, are stable and resistant to degeneration during processing and storage [9].

A unique advantage of phospholipids as a biomarker is the capacity to normalize samples by total or individual phospholipid content, in addition to using internal standards for quantitation. Quantitation is a process of sequential normalization of measured peak intensities of metabolites of interest to the peak intensities of exogenous internal standards, or to a response curve generated using standard materials and exogenous internal standards [18,28]. This is the commonly used method to calculate the concentrations of metabolites. The use of an exogenous internal standard through the calculation of relative ratios significantly reduces variations generated during sample preparation and analysis. However, sample amount variations and inherent variations due to pipet errors cannot be corrected by the use of an internal standard, which have a more significant interference than analytical variations [28]. Variations in sample quantity through provider collection variation and pipet errors can be corrected by the use of endogenous internal standards, which will also correct analytical variations.

In the current study, we used the ratio of two related phospholipids species, where one species serves as an endogenous internal standard. This is integral as phospholipids are uniquely regulated in both dependent and independent pathways. Relative phospholipid quantification allows the absolute quantity to be normalized against each sample’s contributing phospholipids, decreasing the impact of sample collection variation on biomarker results. This provides practical utility when analyzing clinical samples, which can carry significant variation in the amount of sample collected. As such, quantitation of phospholipid biomarkers in clinical practice should be improved through biomarkers, which implement a relative ratio.

We found that implementing this ratio significantly reduced the individual species variations when compared to the concentrations of individual species. Our results emphasize that utilization of ratios between phospholipid species greatly outperforms quantitation individual species in the identification of potential ovarian cancer biomarkers, clearly demonstrating the strength of using a relative ratio over absolute phospholipid content. Furthermore, the use of an endogenous standard via a phospholipid ratio removes the potential for interprovider variation on sample collection techniques. This removes a significant clinical variable, which has impacted previous attempts at developing a phospholipid based ovarian cancer [11]. This analysis represents one of the first investigations to identify an ovarian cancer biomarker with the potential to distinguish adnexal mass etiology with clinically relevant sensitivities and specificity.

In the current study, we limited our analysis to pairing phospholipids sharing the same head group and similar mass responses. However, molar ratios based on quantitation of individual species or peak ratios based on completely characterized mass responses will allow for mixed-class analysis, further improving on the potential power of biomarker selection using this methodology. Regardless of the application, development of a standard mixture targeting all phospholipid species of interest will significantly enhance the performance of phospholipids by reducing batch-to-batch and even mass machine-to-machine variations. The capacity to tailor quantitation methodologies further demonstrates the unique potential of phospholipids as biomarkers.

A limitation in our study is that further patient demographic information was unable to be obtained from de-identified sources, as such future work should incorporate potentially confounding variables such as menstruation status, age variation, iatrogenic estrogen or progesterone use, smoking status, and past medical conditions such as heart failure. Furthermore, the limited sample number and selection algorithm increase the risk for overfitting. Future studies are required validating the identified biomarkers in a separate unique patient population. Future work would also benefit from increased sample sizes to accommodate for the wide variety in benign and cancerous ovarian conditions. In spite of these variations, which might have impacted phospholipids profiles in our samples, we clearly demonstrated that the use of ratios significantly enhanced the diagnostic potential of phospholipids in detecting ovarian cancer.

## 4. Materials and Methods

### 4.1. Materials and Reagents

Reagent-grade chemicals and HPLC-grade solvents were purchased from major commercial suppliers (Fisher Scientific, Hampton, NH, USA, and Sigma Aldrich, St. Louis, MO, USA). 1,2-Dipalmitoyl-sn-glycero-3-phospho-N-methylethanolamine (PME) was purchased from Santa Cruz Biotech (Santa Cruz, CA, USA), and 1-(10Z-heptadecenoyl)-2-hydroxy-sn-glycero-3-phosphocholine (LPC(17:1)), 1-(10Z-heptadecenoyl)-sn-glycero-3-phosphoethanolamine (LPE(17:1)), 1-(10Z-heptadecenoyl)-2-hydroxy-sn-glycero-3-phospho-(1’-myo-inositol) (LPI(17:1)), and 1-heptadecanoyl-2-hydroxy-sn-glycero-3-phosphate (LPA(17:0)) were purchased from Avanti Polar Lipids (Alabaster, AL, USA). Milli-Q water was used throughout.

### 4.2. Sample Collection and Processing

All plasma samples were collected under protocols approved by the Institutional Review Board at the Feinstein Institute for Medical Research. Informed consent was obtained from all patients and controls. (The protocol # is 10-193A). Plasma samples were collected from three groups, patients with confirmed ovarian cancer (cancer, *n* = 20, 16 from stage III and IV, and 4 from stage I and II, 61.7 ± 8.1 years old), benign ovarian tumors (benign, *n* = 20, 57.8 ± 7.1 years old), and healthy, non-cancer pathology (control, *n* = 22, 56.2 ± 5.8 years old) were used. The plasma was separated from blood specimen collected in EDTA containing tubes using centrifugation and stored at −80 °C until analyzed.

### 4.3. Extraction of Phospholipids

Lipids were extracted from the plasma samples following the published method [29]. Briefly, 50 μL of frozen plasma was extracted with 750 μL of methanol with 0.1 nmol of PME, 0.15 nmol of LPE(17:1), 0.85 nmol of LPC(17:1), 0.45 nmol of LPA(17:0), and 0.1 nmol of LPI(17:1) as internal standards. The mixture was vortexed for 2 min, incubated for 10 min at 4 °C, and centrifuged for 10 min at 16,000 g. The supernatant was evaporated to dryness under N_2_. The residue was reconstituted in a 100 μL of solution containing isopropanol (IPA): t-butyl methyl ether (TBME): aqueous ammonium formate (94 mM) (34:17:5, *v*:*v*:*v*). Finally, 20 μL of the solution was injected into the HPLC-MS.

### 4.4. HPLC-MS Analysis

The phospholipid mixture was analyzed using normal-phase HPLC-MS [16,30]. Eluent A was created using IPA: TBME: aqueous ammonium formate (94 mM, pH ~2.5) (34:17:5, *v*:*v*:*v*) with eluent B containing 100% MeOH. The gradients used for the 35 min chromatogram were as follows: 100% A for 18 min, 100% A to 20% A over 6 min, 20% A for 3 min, 20% A to 100% A over 1 min, and hold 100% A for 7 min. The flow rate was 0.3 mL/min and the column temperature was 30 °C. MS and MS/MS data were obtained with an LTQ XL spectrometer (Thermo Scientific, San Jose, CA, USA) operated in the negative ion mode. The full scan MS data was acquired from 180–2000 m/z. The source parameters were sheath gas flow (8 U), spray voltage (4 kV), capillary temperature (300 °C), and capillary voltage (−9 V). MS/MS scan was on the most intense ion in the full-scan spectrum with collision energy of 35 eV. We also performed MS/MS/MS for detailed structural characterization for selected species for detailed structural analysis.

### 4.5. Data Analysis

Obtained data was processed using Thermo X-calibur software (version 2.2) [31]. Retention time and MS and MS/MS data were compared to the control to identify individual species [18]. The concentration of PE and PC includes plasmalogens [30]. PE and PC plasmalogens were denoted as PEP and PCP, respectively. Species between diacyl PE and PEP were distinguished based on their molecular weights and fragmentation patterns by MS/MS. The peak areas of individual species were calculated using M0 and M1 peaks.

### 4.6. Peak Normalization

We first excluded species whose levels changed during 4 h of incubation at room temperature or storage in freezer from further analysis. The incubation at room temperature for 4 h is chosen because clinical samples processed within 4 h of blood drawing are commonly used. After selecting stable and abundant species, we tried three different approaches for peak normalization. The whole procedure is summarized in the flow chart (Figure S4). Firstly, we used peak areas normalized to the internal standards, which calculates concentrations of individual species [18]. For this study, we performed pseudo-quantitation by normalizing lysophospholipids species to corresponding internal standards, LPE (17:1), LPC (17:1), or LPI (17:1), and phospholipids species to PME. Secondly, individual species were normalized to the total content of the class of phospholipids or lysophospholipids. Finally, individual species were normalized to another species within the same phospholipid class.

### 4.7. Statistical Analysis

Data were expressed as the mean ± standard deviation (SD) for continuous variables. The ratios of phospholipids and lysophospholipids were compared using the Mann–Whitney U test for continuous variables, as appropriate. Receiver operating characteristic (ROC) analysis was performed and the area under the curve (AUC) was calculated for factors of the ratios of phospholipids and lysophospholipids in order to assess the accuracy of prediction of ovarian cancer for each factor. To test the stability of phospholipid and lysophospholipids species, the effect of storage time and incubation time on the phospholipid levels was examined using regression analysis with linear regression and Spearman’s correlation coefficients. The *p* values of less than 0.05 were considered to be statistically significant. All analyses were performed using the SPSS software package (version 25.0 J SPSS). Heat map representation of phospholipid profiles in the control, benign, and cancer was generated by Metabo Analyst software v.4.0 [32].

## 5. Conclusions

We showed the potential of plasma phospholipids as a diagnostic marker for ovarian cancer. By using the relative ratio of phospholipid species, we found multiple biomarkers that have high sensitivity and specificity for distinguishing between control, benign, and cancerous ovarian masses. This methodology does not rely on prior protein-based assays but rather uses lipidomics, which is an efficient and promising approach towards ovarian cancer detection.

## Figures and Tables

**Table 1:** The content of phospholipids, lysophospholipids, and sphingomyelin by normalizing the intensities of individual peak areas to the areas of internal standards.

Control vs. Benign			Control vs. Cancer			Benign vs. Cancer		
Species	Ben/Con	*p*	Species	Can/Con	*p*	Species	Can/Ben	*p*
LPE (o-16:0)	0.368	<0.001	PE (16:0/22:6)	3.155	<0.001	PI (18:1/20:4)	0.718	0.014
PE1 (6:0/22:6)	1.832	<0.001	PE (18:0/22:6)	2.606	<0.001	PE (16:0/18:1)	1.544	0.016
PI (18:0/22:6)	1.889	0.001	LPE (22:6)	1.822	<0.001	PI (18:0/20:4)	0.787	0.040
PE (16:0/22:6)	1.774	0.001	LPC (22:6)	2.276	<0.001	PI (16:0/18:1)	0.693	0.042
LPC (18:0)	0.661	0.001	PC (18:0/22:6)	1.754	<0.001	LPC (22:6)	1.460	0.055
PC (16:0/22:6)	1.779	0.001	PC (16:0/22:6)	1.779	<0.001	SM (d18:1/14:0)	0.724	0.055
LPE (18:1)	0.621	0.001	LPE (18:2)	0.648	0.001	PI (16:0/20:4)	0.746	0.055
PE (o-18:0/22:6)	1.889	0.001	LPE (18:1)	0.618	0.001	SM (d18:1/23:0)	0.835	0.066

**Table 2:** The content of phospholipids, lysophospholipids, and sphingomyelin by normalizing the intensities of individual peak areas to the total content of phospholipids.

Control vs. Benign			Control vs. Cancer			Benign vs. Cancer		
Species	Ben/Con	*p*	Species	Can/Con	*p*	Species	Can/Ben	*p*
LPE (20:4)	1.551	<0.001	LPC (20:4)	2.086	<0.001	SM (d18:1/24:1)	1.196	<0.001
LPE (22:6)	2.134	<0.001	PC (18:0/18:1)	0.771	<0.001	SM (d18:1/22:0)	0.873	0.001
LPE (22:5)	1.659	<0.001	LPE (22:6)	2.168	<0.001	PC (18:0/18:1)	0.888	0.002
PC (18:0/20:4)	1.321	<0.001	LPC (22:6)	2.758	<0.001	SM (d18:1/14:0)	0.790	0.002
LPC (22:6)	1.907	<0.001	LPC (18:0)	0.811	<0.001	LPE (18:2)	0.718	0.002
PE (o-18:0/22:6)	1.327	<0.001	LPC (22:5)	2.330	<0.001	PE (16:0/18:1)	1.434	0.006
LPC (20:4)	1.706	<0.001	SM (d18:1/24:1)	1.261	<0.001	SM (d18:2/22:0)	0.903	0.007
PC (o-18:0/20:4)	1.322	<0.001	SM(d18:1/20:0)	0.819	<0.001	SM(d18:1/23:0)	0.866	0.008
PI (18:0/22:6)	1.566	0.001	PC (16:0/20:4)	1.420	<0.001	PE (o-18:0/18:2)	0.732	0.008
PI (18:0/22:5)	1.382	0.001	PE (16:0/22:6)	2.182	<0.001	SM(d18:1/16:0)	1.050	0.010
PE (o-16:0/22:6)	1.302	0.001	PC (o-18:0/20:4)	1.353	<0.001	PE (o-16:0/18:1)	0.780	0.013
PC (18:0/22:6)	1.382	0.002	SM(d18:1/22:0)	0.840	<0.001	LPC (22:6)	1.446	0.014
LPC (18:0)	0.884	0.002	PE (18:0/22:6)	1.939	<0.001	SM (d18:1/20:0)	0.880	0.016
PE (18:0/18:1)	0.633	0.002	PI (18:0/22:6)	1.684	<0.001	PE (o-16:0/18:2)	0.725	0.020
PC (16:0/20:4)	1.233	0.002	LPE (20:4)	1.370	<0.001	PE (16:0/22:6)	1.467	0.023
PC (18:0/18:1)	0.868	0.002	LPE (22:5)	1.589	<0.001	PC (16:0/16:0)	1.150	0.030
PE (16:0/22:6)	1.487	0.003	SM (d18:1/23:0)	0.853	<0.001	LPC (18:0)	0.917	0.033
PI (18:0/20:4)	1.178	0.003	PC (16:0/22:6)	1.602	<0.001	PC (16:0/20:4)	1.151	0.035
LPC (22:5)	1.823	0.003	SM (d18:1/14:0)	0.764	<0.001	PE (o-16:0/22:6)	0.933	0.035

**Table 3:** The area under curves (AUCs) for phospholipids, lysophospholipids, sphingomyelin, their 95% confidence intervals (CI), *p* values, sensitivities, specificities, and cutoff values for (**a**) control vs. benign, (**b**) control vs. cancer, and (**c**) benign vs. cancer.

**(a) Control vs. Benign**	**PC (18:0/20:4)/PC (18:0/18:1)**	**LPE (22:6)/LPE (o-16:0)**	**LPC (22:6)/LPC (18:0)**	**LPC (20:4)/LPC (18:0)**	**PC (18:0/22:6)/PC (18:0/18:1)**
AUC	0.87	0.86	0.85	0.84	0.83
*p* value	<0.001	<0.001	<0.001	<0.001	<0.001
95%CI	0.77–0.98	0.74–0.97	0.72–0.97	0.72–0.96	0.71–0.96
Sensitivity (%)	95	100	80	65	90
Specificity (%)	73	64	86	91	68
Cutoff Value	2.12	3.9	0.07	0.37	0.35
**(b) Control vs. Cancer**	**LPC (20:4)/LPC (18:0)**	**LPC (22:6)/LPC (18:0)**	**LPC (20:4)/LPC (16:0)**	**LPC (22:6)/LPC (o-16:0)**	**SM (d18:1/24:1)/SM (d18:1/22:0)**
AUC	0.95	0.94	0.94	0.92	0.92
*p* value	<0.001	<0.001	<0.001	<0.001	<0.001
95%CI	0.89–1.00	0.88–1.00	0.87–1.00	0.84–1.00	0.83–1.00
Sensitivity (%)	90	95	80	95	100
Specificity (%)	91	82	96	82	73
Cutoff Value	0.37	0.07	0.14	4	1.13
**(c) Benign vs. Cancer**	**SM (d18:1/24:1)/SM (d18:1/22:0)**	**SM (d18:1/16:0)/SM (d18:1/22:0)**	**SM (d18:1/16:0)/SM (d18:1/14:0)**	**SM (d18:1/24:1)/SM (d18:1/14:0)**	**PE (16:0/18:1)/PE (o-18:0/18:2)**
AUC	0.84	0.82	0.82	0.81	0.77
*p* value	<0.001	<0.001	0.001	0.001	0.003
95%CI	0.71–0.96	0.70–0.95	0.68–0.96	0.66–0.95	0.62–0.92
Sensitivity (%)	90	90	80	80	55
Specificity (%)	70	65	80	85	100
Cutoff Value	1.23	2.41	11.3	6.24	0.47

## Supplementary Materials

The following are available online at https://www.mdpi.com/2072-6694/12/1/72/s1. Figure S1: The total ion chromatogram and MS spectra of plasma phospholipids, Figure S2: The MS/MS/MS spectra of SM (d18:1/16:0) and SM (d18:2/18:0), Figure S3: Changes in the ratio of phospholipids depending on sample amount, Figure S4: Flow chart for study design and biomarker selection procedure.
