# Supplementary material for: Relative Ratios Enhance the Diagnostic Power of Phospholipids in Distinguishing Benign and Cancerous Ovarian Masses
Source: Cancers (Basel). 2019 Dec 26;12(1):72. doi: 10.3390/cancers12010072 (PMC7016589; doi:10.3390/cancers12010072)
Supplement: Supplementary file 1 [file cancers-12-00072-s001.pdf]

Supplementary Materials

Relative Ratios Enhance the Diagnostic Power of Phospholipids in Distinguishing Benign and Cancerous Ovarian Masses

Tsukasa Yagi, Cyrus E. Kuschner, Muhammad Shoaib, Rishabh C. Choudhary, Lance, B, Becke, Annette T. Lee, Junhwan Kim

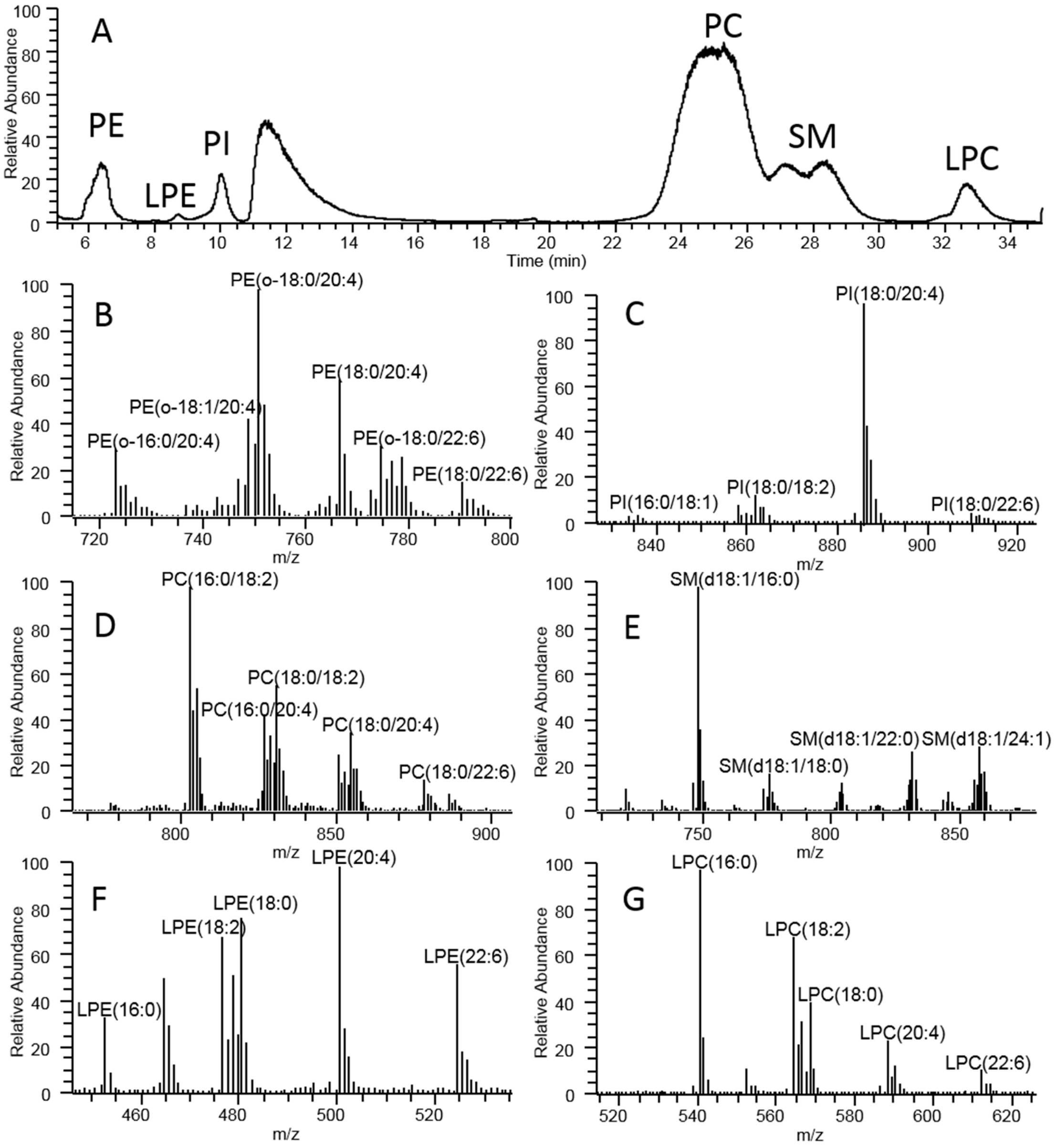

Figure S1. The total ion chromatogram and MS spectra of plasma phospholipids. HPLC-MS analysis (**A**) and MS spectra of phosphatidylethanolamine (PE) (**B**), phosphatidylinositol (PI) (**C**), phosphatidylcholine (PC) (**D**), sphingomyelin (SM) (**E**), lysophosphatidylethanolamine (LPE) (**F**), and lysophosphatidylcholine (LPC) (**G**).

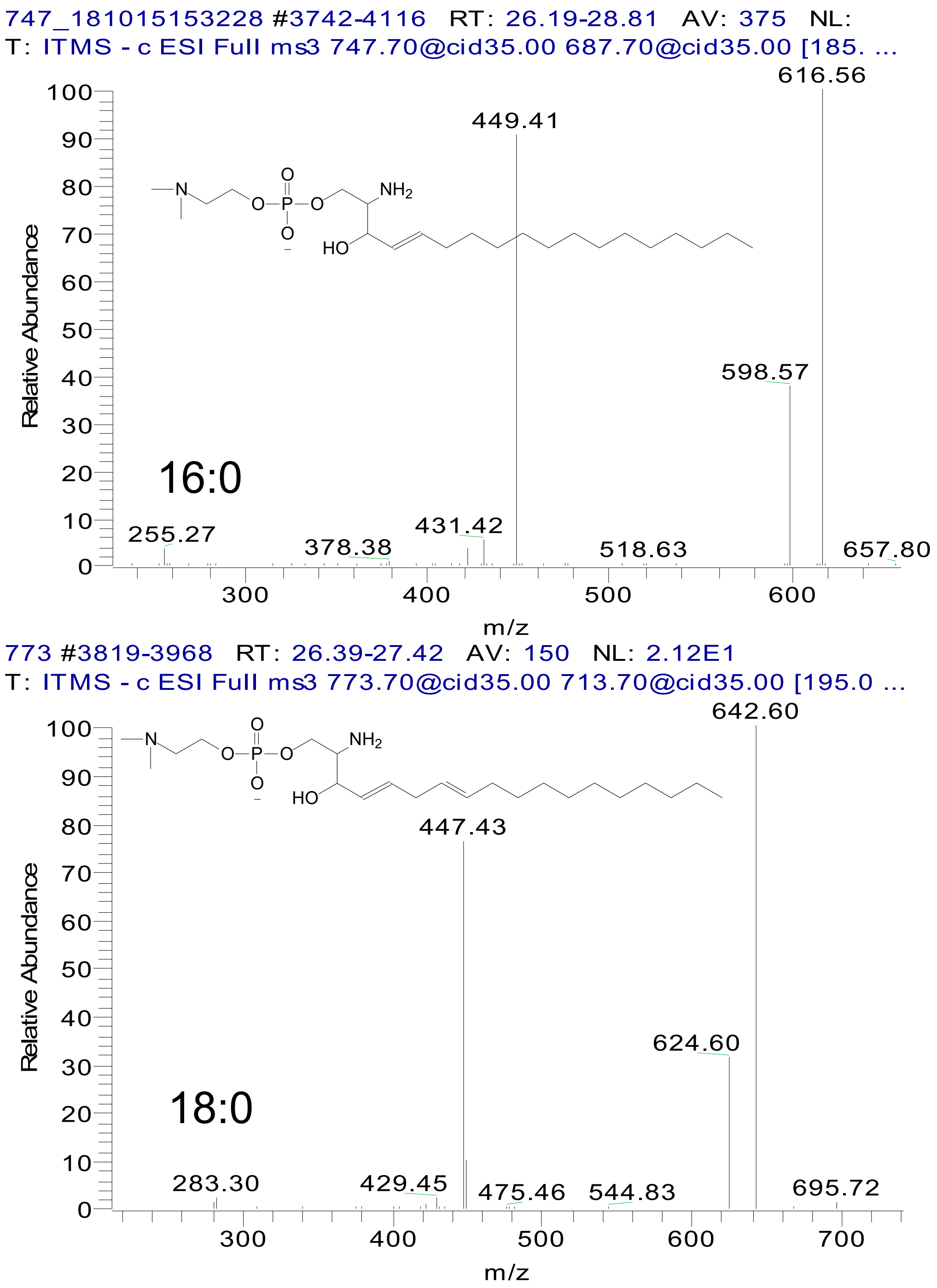

Figure S2. The MS/MS/MS spectra of SM(d18:1/16:0) and SM(d18:2/18:0). The peaks at 449.4 and 255.3 from SM(d18:1/16:0) (top panel) and peaks at 447.7 and 283.3 from SM(d18:2/18:0) (bottom panel) confirms the structure as proposed. The position of the second double bond of SM(d18:2/18:0) is not determined.

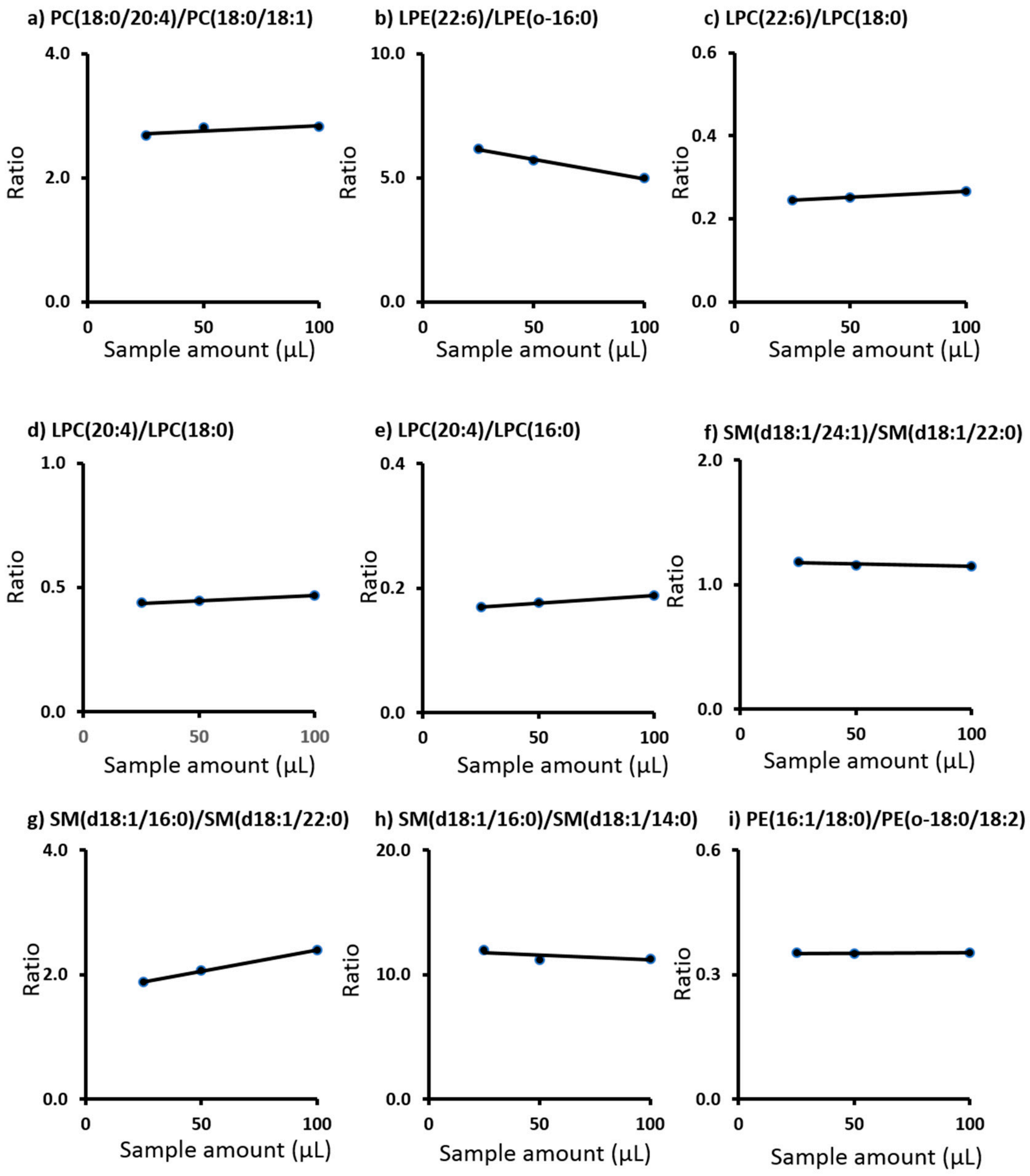

Figure S3. Changes in the ratio of phospholipids depending on sample amount. The ratios of phospholipids do not change significantly when analyzed in 25 µL, 50 µL, or 100 µL of plasma.

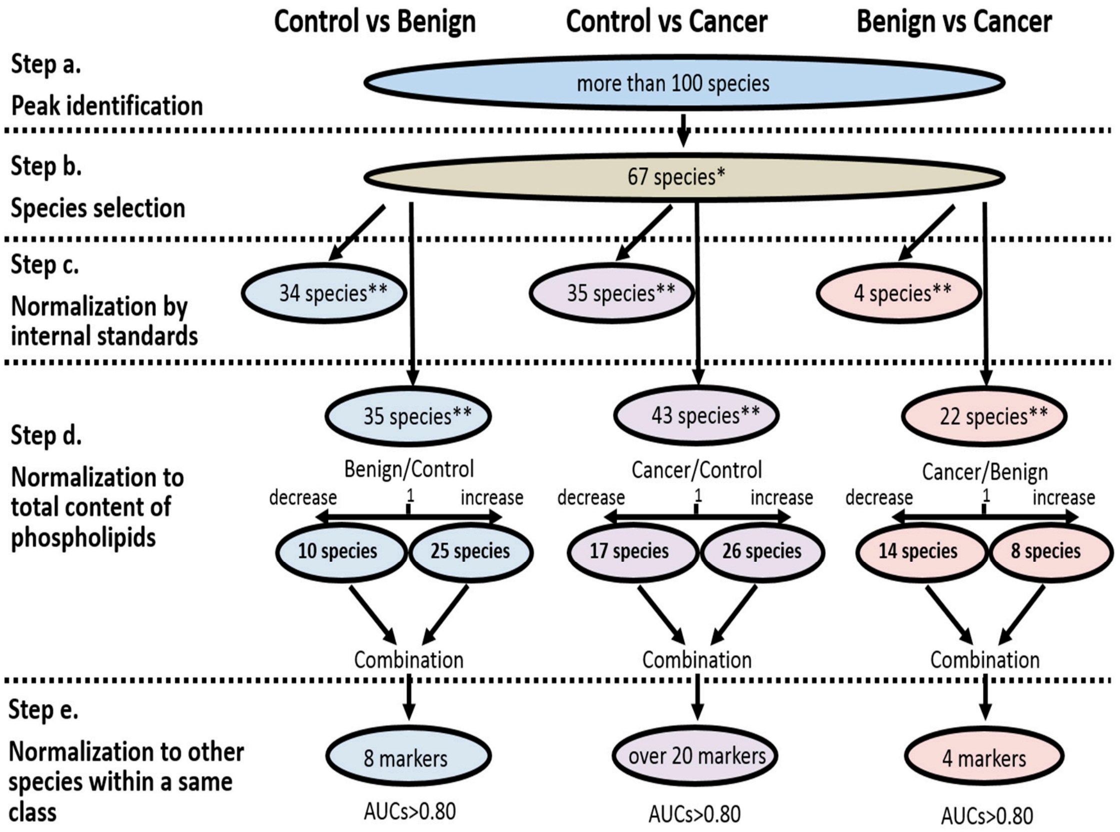

Figure S4. Flow chart for study design and biomarker selection procedure. Unstable phospholipid species and minor species were excluded from analysis (*, quantifiable and stable species; **, significantly different between the two group (p < 0.05); cancer, n = 20; benign ovarian tumors (benign), n = 20; and healthy, non-cancer pathology (control), n = 22).

© 2019 by the authors. Licensee MDPI, Basel, Switzerland. This article is an open access article distributed under the terms and conditions of the Creative Commons Attribution (CC BY) license (http://creativecommons.org/licenses/by/4.0/).
